# Socio-demographic Correlates of Fathers' and Mothers’ Parenting Behaviors

**DOI:** 10.1007/s10826-018-1059-7

**Published:** 2018-03-22

**Authors:** Jacobien Van Holland De Graaf, Marcel Hoogenboom, Simone De Roos, Freek Bucx

**Affiliations:** 1 Zeist Municipality, Zeist, The Netherlands; 2 Faculty of Social and Behavioral Sciences, Utrecht University, Heidelberglaan 1, 3584 CS Utrecht, The Netherlands; 3 The Netherlands Institute for Social Research (SCP), The Hague, The Netherlands

**Keywords:** Parenting behavior, Support, Control, Fathers, Mothers

## Abstract

In the present study, we investigated whether fathers’ and mothers’ parenting behavior is differentially related to parental factors (such as age and employment), child factors (age and gender) as well as social support. Parents reported on their use of a broad range of parenting behaviors, including affection, responsivity, explaining, autonomy, support, rewarding, and punishing. We used survey data from the Netherlands for 1197 mothers and 903 fathers of children aged 2 to 17. Seemingly unrelated regression analyses were conducted to combine the regression results on the separate subsamples (fathers and mothers) and to test for differences in the coefficients between those subsamples. Our expectation that the parenting behavior of fathers is more dependent on parents’ characteristics, children’s characteristics, and social support than that of mothers was only partly confirmed by the results of our analysis. In general, our results suggest that fathers’ parenting behaviors seem to be associated with parental and child characteristics and contextual factors in ways that are similar to how these factors are associated with mothers’ parenting behaviors. Results are discussed in relation to the roles and expectations associated with motherhood and fatherhood.

## Introduction

Societal changes have profoundly affected family life in Western countries over the past 40 years. Especially rising divorce rates and mothers’ participation in the labor force have stimulated mothers and fathers to reorganize their family life, with fathers taking a more prominent role in parenting (Lamb 2010). Mothers still spend considerably more time with their children than fathers, but research shows that in many Western countries the amount of time that fathers and mothers allocate to their children is converging (Gauthier et al. 2004; Raley et al. 2012). Nowadays fathers tend to play a more active role in the parenting of their children (Cabrera and Tamis-LeMonda 2013; Lamb 2010; Planalp and Braungart-Rieker 2016), although there is a wide variation in fathers’ involvement too (Amato et al. 2009). The Netherlands, where our study was conducted, is illustrative of a country that has witnessed the aforementioned societal changes, including increases in divorce and female employment (Merens and Van den Brakel 2014).

Besides the time they spend with their children, further evidence suggests that fathers and mothers are also becoming more similar in terms of their parenting roles and behaviors and the way in which their behaviors affect children’s adjustment (Fagan et al. 2014). Although the relative frequency of specific behaviors might be different, research suggests that the behavioral strategies that fathers and mothers undertake in order to support or discipline their children are generally similar (Adamsons and Buehler 2007; Asbourne et al. 2011; Van Leeuwen and Vermulst 2004). For example, it has been found that fathers and mothers interact with their young children with equal sensitivity (Notaro and Volling 1999). This is why some researchers claim that at present there are more similarities than dissimilarities in how mothers and fathers interact with their children (Kennedy et al. 2015; Lamb 2010).

Mothers’ and fathers’ parenting does not take place in a vacuum, instead it is influenced by multiple intrafamilial and extrafamilial factors. This is an important assumption in Belsky’s (1984; see also Belsky and Jaffee 2006) “process model” of competent parenting, which also specifies the interplay between parent and child. Comparable ideas underlie Bronfenbrenner’s “ecological systems theory” (Bronfenbrenner and Luscher 1995), which focuses on the intersecting, multiple systems in which children and parents are embedded. Both models have been widely used to examine several aspects of the parenting process, including parent–child attachment and other developmental outcomes, parental involvement, intergenerational transmission of supportive parenting, and determinants in research on parenting (Cabrera et al. 2014). The models stress the influence of parental factors, child factors and family contextual factors on the parenting process. Thusfar, it is not clear whether the influence of these factors on parenting differs for mothers and fathers. Before we theorize as to why there might be differences between mothers and fathers in correlates of parenting, we describe several parental and child factors as well as social support aspects that have been studied as possible correlates of parenting behavior.

Regarding *parental factors*, several characteristics like parental age, education and occupation have been found to be resources that may contribute to how parents rear their children (Cabrera et al. 2014; Castillo et al. 2011). Research suggests that the age of parents and their parenting behavior are related: the older the parents are, the more emotional stability and self-control they tend to develop, the greater their involvement, and the better they appear able to cope with the stresses of parenthood (Castillo et al. 2011; Verhoeven et al. 2007). The educational level of the parents may also contribute to differences in parental behavior (Bradley and Corwyn 2002; Gracia 2015; Trifan et al. 2014). During their education, higher-educated parents might have learned to reflect on their own behavior, while in their occupational life they may be more stimulated and/or forced to make their own decisions (Cabrera et al. 2014).

Besides parents’ educational level and age, number of working hours can be a factor determining parenting behavior. With the growing participation of women in the labor market, reconciliation of labor and care has now become a more topical issue. The time parents can dedicate to parenting is thus getting increasing attention in scientific research (Raley et al. 2012; Roeters et al. 2009), possibly, as Crouter and McHale (2005) suggested, as a result of worries about the risks for children when parents—more specifically women—work more hours. Still, research showed that a mother’s number of working hours does not affect her knowledge of her children’s daily activities or the time she spends with them (Bianchi 2000; Roeters et al. 2009). Working mothers may spend more time with their children before school and on off-work days than non-working mothers (Crouter and McHale 2005). For fathers it was found that they spend less time on both routine and interactive activities with their child when they work more hours (Roeters et al. 2009).

Parenting is not a one-way street: parenting behavior influences a child’s development, but certain *child factors* may influence parenting strategies (Lam et al. 2013). One such characteristic is the gender of the child. Some studies showed the use of different parenting styles for sons and daughters (for example: Leaper 2002; McKinney and Renk 2008). Leaper (2002) suggested that, deliberately or inadvertently, parents often treat girls and boys differently, as once they grow up they are expected to take up different roles in society. Parenting behavior can also vary depending on the child’s age. For example, parents spend less time with their children, know less about their daily activities, or grant higher levels of autonomy as the child grows older (Hofferth et al. 2007; Lam et al. 2013; Phares et al. 2009; Roche et al. 2014; Verhoeven et al. 2012). Research also showed that parents perceive themselves as more effective with a same-gender child, possibly because role identification with an opposite-gender child is more difficult (Grolnick et al. 1996). With a same-gender child it may be easier to undertake activities or to recognize the needs of the child because of the parent’s own experiences. Both mothers and fathers have been found to spend more time with children of their own gender (Lam et al. 2013).

Also, *family contextual factors* may affect parenting behavior, for example persons and institutions outside the nuclear family (McConnell et al. 2011). Receiving social support from family members and friends (informal support) or institutions like childcare, playgroups or school (formal support)—conceptualized in our study as discussing parenting and receiving advice—can help parents cope with the challenges and difficulties of bringing up a child (Cochran and Walker 2005; Kesselring et al. 2012). Positive relations have been found for support from family and friends, and for fathers’ involvement with their children (Castillo and Frenzl-Crossman 2010). Perceived support was found to be positively related to parental warmth and monitoring for mothers and fathers of Mexican origin (Taylor et al. 2015). Less support from relatives was linked to inadequate supervision in low-income mothers (Coohey 2007).

From a theoretical viewpoint, there are several reasons why fathers’ more than mothers’ parenting behavior might be dependent on specific situations or circumstances (see, for example, Eagly and Wood 1999; Elam et al. 2017; Katz-Wise et al. 2010). First, women have a greater biological role in childbearing than men. A mother’s nurturing relationship with her children may be rooted in the biological conditions associated with pregnancy, birth and breastfeeding (Eagly and Wood 1999). Second, the role of motherhood for women is associated with different societal expectations than the role of fatherhood for men, and there are societal pressures to conform to these roles. Many cultures are still quite specific about women being responsible for parenting, much more so than men (Bianchi et al. 2012). Women tend to receive extensive socialization for the parenting role, while men are relatively less prepared for parenthood (Parke and Brott 1999). In most Western countries the majority of women are still the primary caregiver (Bianchi et al. 2012). This also applies to the Netherlands, where the vast majority of adult women work part-time (Merens and Van den Brakel 2014), predominantly because they feel that they have to take care of their young children (Kremer 2006). Third, though it is now well-established in the literature that both women and men have the capacity to be good parents and most parenting skills are learned by doing, mothers spend on average more time on parenting than fathers (Lamb 2012) and are more invested in the parenting role (Elam et al. 2017). Partially as a result, mothers also tend to take up more responsibility for management family tasks, like setting boundaries for the child and arranging daycare (Parke 2000).

Considering these differences, parenthood has been found to be more salient for women’s identity than for men’s, and women are more likely to feel an obligation or duty to engage in parenting than men (Katz-Wise et al. 2010; Simons et al. 1990). Whereas women thus feel a limit in how much they can withdraw from parenting, men may experience much more freedom in their parental role (Simons et al. 1990). Hence men may have more discretion in defining their caring role than women, leading to more variation in how fathers fulfill their parenting role (Roeters et al. 2009). Consequently, there is reason to expect parental factors, child factors, and social support to exert more influence on the parenting practices of fathers than of mothers (Elam et al. 2017).

Thus far, however, results from previous research are inconclusive as to whether the influence of these multiple intrafamilial and extrafamilial factors on parenting differs for fathers and mothers. Some studies found the same correlates for both maternal and paternal behavior (Castillo et al. 2011; Lam et al. 2013; Lickenbrock and Braungart-Rieker 2015; Trifan et al. 2014; Verhoeven et al. 2007), whereas others showed differences between fathers and mothers (Elam et al. 2017; Grolnick et al. 1996; Verhoeven et al. 2012). With respect to parental factors, fathers were generally more strongly influenced by their educational level and partner’s work demands than mothers (Grolnick et al. 1996; Roeters et al. 2009). More mixed findings were found on child factors: age and gender of the child were found to be related to the parenting behavior of mothers only (Verhoeven et al. 2012), to the parenting behavior of both fathers and mothers (Lam et al. 2013), or to that of fathers only (Chaplin et al. 2005; Wright et al. 2013). Regarding contextual factors, social support has been found to be related to parental behavior of both mothers and fathers, but so far it remains unclear whether this relation is stronger for fathers than for mothers.

More empirical research is therefore needed to demonstrate the relative importance of specific factors in the same type of parenting behavior. An important prerequisite is that studies use the same measures to assess parenting of both fathers and mothers—a condition met by many but not all studies (see, for example, Fagan et al. 2014). In addition, many studies on parenting correlates focused mainly on the time parents spend on parenting, or alternatively examined only a restricted range of parenting behaviors (Marsiglio et al. 2000; Planalp and Braungart-Rieker 2016).

In the present study we investigated whether fathers’ and mothers’ parenting behavior is differentially related to parental factors, child factors, and social support. Based on the theoretical arguments we presented, these factors may be expected to be associated more with the parenting practices of fathers than those of mothers. We examined a broad range of parenting strategies, doing justice to the multidimensional nature of parenting, and assessed the same parenting constructs for both fathers and mothers. The following hypotheses were formulated. First, for parental factors we expected educational level (Hypothesis 1a), age (Hypothesis 1b), parent’s number of working hours (Hypothesis 1c) and partner’s number of working hours (Hypothesis 1d) to be stronger predictors of the parenting behavior of fathers than of the parenting behavior of mothers. Second, for child factors we expected gender (Hypothesis 2a) and age of the child (Hypothesis 2b) to be stronger predictors of the parenting behavior of fathers than of the parenting behavior of mothers. Finally, for social support we expected the social support that parents receive to be a stronger predictor of the parenting behavior of fathers than of the parenting behavior of mothers (Hypothesis 3).

## Method

### Participants

The data in this study were drawn from the “Parenting in the Netherlands 2010” study (Opvoeden in Nederland 2010), a large-scale study of parenting in a random address sample of 2827 households with at least one parent and one child aged 0–17. For the current study we selected heterosexual parents living with a partner (85.8% of the total sample) whose child living at home was between ages 2 and 17 (86.6% of the resulting sample). The resulting sample included 2100 respondents, 903 (43.0%) fathers and 1197 (57.0%) mothers.

### Procedure

The data were collected in 2010. Parents were asked to fill out a questionnaire online or a printed version. Within each sampled household with two parents, one parent was randomly selected to fill out the questionnaire. In households with more children, one child from the household was randomly selected to be the study reference child, and was referred to throughout the questionnaire. The overall response rate was 38%—about average for family studies in the Netherlands (Dykstra et al. 2005). Weighted descriptive analyses were used, correcting for underrepresentation of parents with lower incomes and lower education, to adjust our parent sample to the composition of the Dutch population of parents with children living at home.

### Measures

#### Parenting strategies

Mothers’ and fathers’ parenting behaviors were investigated with six scales, each representing a different parenting strategy. For each scale, statements were evaluated on a 6-point scale, varying from 0 = completely disagree to 5 = completely agree. All parenting scales were based on subscales from the Dutch Parenting Questionnaire (Nijmeegse Opvoedingsvragenlijst; Gerris et al. 1993), except for the scale that measured explaining, which was based on the work of Peeters and Woldringh (1993). These scales represent instruments previously validated in research in the Netherlands (Rispens et al. 1996). Below we describe each scale in more detail.

Two parenting strategies were measured that relate to the support parents provide to children, namely affection and responsivity. The affection scale (five items) measured the extent to which parents indicate using perceptible expressions of positive affection and devotion in parenting. An example of an item was: “I often cuddle, kiss, and hug my child”. The reliability of the scale was high (*α* = 0.80 for mothers’ reports, and 0.84 for fathers’ reports). The scale used to measure responsivity is composed of six items and attempts to determine the extent to which parents are responsive to needs, signals, and the condition of the child. An example of an item was: “I know exactly when my child is having a difficult time”. The reliability of the scale was found to be high (*α* = 0.83 for mothers’ reports, and 0.87 for fathers’ reports).

Four parenting strategies were measured that relate to the use of control: explaining, autonomy support, rewarding, and punishing. The scale measuring explaining (five items) concerns the extent to which parents provide their children with instructions about how to behave and explanations for why specific behaviors are unwanted. An example of an item was: “If I ask my child to do something in the family, I first provide him/her with clear instructions”. The reliability of this scale was reasonable (*α* = 0.65 for mothers’ reports, and 0.64 for fathers’ reports). The autonomy support scale (six items) measured the extent to which parents try to encourage and stress the child’s independence and sense of responsibility for its actions, thoughts, and decisions. An example of an item was: “I often let my child make his/her own decisions”. With *α* = 0.73 for mothers’ reports and 0.70 for fathers’ reports, the scale’s *α* was found to be good. The rewarding scale (five items) measured the extent to which parents indicate rewarding their child materially for good behavior or try to guide the child’s behavior by showing approval. An example of an item was: “I often give my child a compliment when he/she has assisted me with something”. With *α* = 0.75 for mothers’ reports and 0.73 for fathers’ reports, the scale’s *α* was found to be good. The punishing scale (six items) determined the extent to which parents apply sanctions when the child misbehaves. An example of an item was: “I often punish my child by denying him/her something nice”. The reliability of this scale was reasonable (*α* = 0.68 for mothers’ reports, and 0.69 for fathers’ reports).

#### Parental and child characteristics, and social support

Parents reported on their age (in years) and their educational level, ranging from: 1 (=none) to 8 (=tertiary education, university). For education it was necessary to construct an appropriate interval scale: we applied a standard recoding procedure whereby the original categories were transformed into new categories defined by the approximate number of years of education completed (De Graaf et al. 2000). The resulting variable ranged from 6 = completion of elementary school but not secondary or vocational education to 16 = completion of post-graduate education. The number of working hours of the parent and the partner was measured by first asking respondents whether they or their partner were employed. A continuous variable was created by scoring 0 if the respondent/partner was not employed; if employed, respondents were asked to provide the number of working hours according to their labor contract or, if no contract was available, the actual number of working hours.

Parents reported on their child’s gender (0 = male, 1 = female) and age (in years). Finally, social support was measured by five items in which the respondents were asked how often they had received advice from or discussed parenting with (1) their own parents/parents-in-law, (2) other family members, (3) friends, (4) neighbors, and (5) the people working at their child’s childcare facility, playgroup, or school (derived from Deković et al. 1996). The answer options ranged from 1 (=never) to 6 (=daily). As these five items correspond to different sources of support, they were used as separate predictors in the regression analyses.

### Data Analyses

Findings are reported in three steps. First, we offered brief descriptive information on frequencies of parenting behaviors of fathers and mothers and on intercorrelations among parenting behaviors, parental and child factors, and social support. Second, correlates of parenting behaviors were estimated in separate regression models for fathers and mothers. Third, Stata’s Seemingly Unrelated ESTimation (SUEST) postestimation command was used to combine regression results on the separate subsamples (fathers and mothers) and to test for differences in the coefficients between those subsamples (with a Wald chi-square test) (Weesie 1999). SUEST analyses should give the exact same results as an interaction test with a robust command when gender of the parents is used as an interaction term with all predictors. The advantage of using SUEST is that differences between groups can be tested without complicated interaction variables that increase the risk of multicollinearity between predictor variables. The full models of the subsamples are presented, including reports of statistical significance between the groups’ coefficients.

Prior to the regression analyses, we checked the model assumptions. Because the scores on the parenting scales were skewed, the natural logarithm of these variables were used in additional analyses (Tabachnick and Fidell 2013). As this did not change the results, we retained the original variables for our regression analyses. We also tested the independent variables for multicollinearity with the Variance Inflation Factor (VIF), which for both fathers (VIF ranging from 1.01 to 3.30) and mothers (VIF ranging from 1.00 to 3.80) showed no problems. Finally, we tested whether the measures of parenting behaviors were equivalent (invariant) across fathers and mothers, using the multi-group factor analysis with alignment (Asparouhov and Muthén 2014). Results indicated that, for the great majority of measurement items, comparisons across fathers and mothers were invariant. Where factor loadings or intercepts of items were non-invariant, we performed additional analyses on scales in which these items were excluded. Because results were not changed, we decided to report the regressions based on the original measurement scales. Further information on these additional analyses is available upon request from the first author.

## Results

### Descriptive Results and Correlations

The descriptive characteristics of the sample are displayed in Table 1. The table indicates that fathers were slightly older than mothers and worked more hours than mothers. Working hours for partners were lower among fathers than mothers. Fathers’ educational level was higher than mothers’. For parenting strategies Table 1 shows no significant differences between scores of fathers and mothers in the extent to which they give their child autonomy, or punish or reward them. Fathers however reported giving significantly less affection to their children than mothers, while also scoring significantly lower on responsivity and explaining. Fathers also reported receiving less support from parents/parents-in-law and from the childcare facility, playground, or school than mothers.

**Table 1:** Descriptive characteristics of the dependent and independent variables for fathers (n = 903) and mothers (n = 1197)

	Fathers				Mothers				
Variables	*M*	SD	Range	*%*	*M*	SD	Range	*%*	*t*
*Parenting behaviors*									
Affection	5.26	.70	1.8–6		5.45	.57	1–6		−6.67***
Responsivity	5.14	.63	2.3–6		5.40	.52	1–6		−10.02***
Explaining	5.13	.59	1.8–6		5.35	.52	1–6		−9.15***
Autonomy support	4.28	.76	1–6		4.34	.75	1.5–6		−1.89
Punishing	3.42	.91	1–5.8		3.43	.90	1–6		−0.33
Rewarding	3.47	.97	1–6		3.42	.99	1–6		1.11
*Parental factors*									
Level of education (years)	13.36	2.24	6–16		13.07	2.15	6–16		2.96**
Age (years)	42.90	6.46	23–60		40.38	6.12	22–60		9.07***
Working hours	35.77	13.24	0–90		17.81	11.93	0–75		−32.10***
Working hours partner	17.69	11.94	0–50		35.62	13.75	0–85		−31.91***
*Child factors*									
Gender									
Male				54				51	
Female				46				49	
Age (years)	9.42	4.67	2–17		9.37	4.55	2–17		0.21
*Social support, from:*									
Parents/parents-in-law	2.66	1.17	1–6		2.93	1.22	1–6		−5.10***
Other family members	2.53	1.01	1–6		2.62	1.05	1–6		−5.86***
Friends	2.71	1.06	1–6		3.11	1.07	1–6		−8.63***
Neighbors	1.76	0.97	1–6		1.79	1.00	1–6		−0.73
Childcare/playgroup/school	2.39	1.08	1–6		2.50	1.05	1–6		−2.39**

For dummy variables only the percentages are reported

*p < .05. **p < .01. ***p < .001

In Table 2, the intercorrelations are shown between the variables of interest. The pattern of correlations between parental and child characteristics, on the one hand, and parenting behavior, on the other hand, is for the most part comparable for fathers and mothers. Fathers’ as well as mothers’ level of education was positively related to autonomy support but negatively related to punishment and rewarding. Additionally, there was a positive correlation between mothers’ level of education and affection. Mothers’ number of working hours was positively related to mothers’ autonomy support and negatively related to punishment. Fathers with partners who work more hours per week showed more affection, explained more often, were higher in autonomy support, but made less use of punishment strategies compared to fathers with partners who work fewer hours. For both fathers’ and mothers’ reports, parent’s age as well as child’s age was positively related to autonomy support, but negatively related to (almost) all other parenting strategies.

**Table 2:** Intercorrelations among parenting behaviors, parental and child factors, and social support

	1	2	3	4	5	6	7	8
*Parenting behaviors*								
1 Affection	_	0.63***	0.55***	0.14***	0.04	0.10**	0.08**	−0.23***
2 Responsivity	0.69***	_	0.62***	0.14***	0.01	0.09**	0.02	−0.13***
3 Explaining	0.52***	0.65***	_	0.25***	0.02	0.10***	0.05	−0.02
4 Autonomy support	0.08*	0.10**	0.23***	_	−0.06*	0.18***	0.16***	0.18***
5 Punishment	0.04	0.02	0.10**	−0.06	_	0.29***	−0.15***	−0.29***
6 Rewarding	0.19***	0.10**	0.11**	0.15***	0.32***	_	−0.07*	−0.16***
*Parental and child factors*								
7 Level of education	−0.04	−0.02	0.01	0.16***	−0.12***	−0.11***	_	0.04
8 Age	−0.27***	−0.16***	−0.10**	0.14***	−0.23***	−0.09*	0.14***	_
9 Working hours	−0.01	−0.02	−0.02	0.00	−0.00	0.04	0.04	−0.13***
10 Working hours partner	0.09**	0.05	0.07*	0.17***	−0.09**	−0.01	0.18***	0.03
11 Child’s age	−0.42***	−0.22***	−0.17***	0.24***	−0.24***	−0.12***	0.02	0.62***
*Social support, from:*								
12 Parents/parents-in-law	0.08*	0.03	0.00	0.00	0.13***	0.10**	0.04	−0.31***
13 Other family members	0.03	0.00	−0.01	0.05	0.08*	0.12***	0.04	−0.09**
14 Friends	0.06	0.00	0.03	0.08*	0.02	0.09**	0.12***	−0.02
15 Neighbors	0.07*	−0.01	0.02	0.03	0.04	0.12***	0.04	−0.08*
16 Childcare/playgroup/ school	0.12***	0.05	0.08*	0.02	0.09*	0.08*	0.05	−0.18***

	9	10	11	12	13	14	15	16
*Parenting behaviors*								
1 Affection	0.02	0.01	−0.32***	0.10***	−0.00	0.03	−0.03	0.12***
2 Responsivity	−0.01	0.01	−0.14***	0.04	−0.04	−0.05	−0.04	0.01
3 Explaining	−0.01	0.01	−0.04	−0.00	−0.00	0.01	−0.05	0.02
4 Autonomy support	0.17***	−0.04	0.23***	−0.04	−0.03	0.05	−0.00	−0.03
5 Punishment	−0.12***	−0.02	−0.23***	0.13***	0.05	0.09**	0.02	0.19***
6 Rewarding	−0.03	−0.04	−0.11***	0.11***	0.05	0.07*	0.01	0.17***
*Parental and child factors*								
7 Level of education	0.26***	0.07*	−0.10***	0.06	0.03	0.11***	0.07*	0.05
8 Age	0.05	−0.02	0.68***	−0.33***	−0.04	−0.01	−0.03	−0.28***
9 Working hours	_	0.03	0.05	0.06	−0.03	0.01	0.02	0.03
10 Working hours partner	0.02	_	−0.02	0.04	−.01	0.04	0.04	−0.00
11 Child’s age	−0.03	−0.05	_	−0.28***	−0.06*	−0.09*	−0.07*	−0.32***
*Social support, from:*								
12 Parents/parents-in-law	0.06	0.06	−0.22***	_	0.39***	0.33***	0.22***	0.31***
13 Other family members	−0.01	0.03	−0.10**	0.52***	_	0.48***	0.29***	0.27***
14 Friends	−0.02	0.04	−0.10**	0.40***	0.56***	_	0.31***	0.36***
15 Neighbors	0.04	0.01	−0.15***	0.31***	0.40***	0.46***	_	0.24***
16 Childcare/playgroup/school	0.02	0.06	−0.30***	0.35***	0.32***	0.41***	0.39***	_

Correlations for fathers are below diagonal; correlations for mothers above diagonal

*p < .05. **p < .01. ***p < .001

As for social support, several correlations were found significant. For both fathers and mothers, there was a positive relationship between affection and support from own parents, from neighbors and from childcare/school. Also, the parents’ provision of punishment was positively correlated with support from own parents, and from childcare/school. Furthermore, fathers’ as well as mothers’ rewarding was positively associated with support from own parents, from friends, and from childcare/school; in addition, fathers’ rewarding was positively related to support from other family members and from neighbors. Finally, there was a positive correlation between fathers’ explaining and support from childcare/school as well as between fathers’ autonomy support and support from friends.

#### Regression models for fathers and mothers

In the second step, correlates of parenting behaviors were estimated in separate regression models for fathers and mothers. The results are presented in Table 3: the second and the fifth column show the results for the fathers, the third and sixth column for the mothers. There were significant and robust relations between parents’ educational level on the one hand, and autonomy support and punishment on the other: the higher the parent’s educational level, the more the child was provided with autonomy, and the less he/she was punished. There were also significant relations between age of the child and affection and autonomy. These results indicate that parents showed less affection to older children and provided them with more autonomy.

**Table 3:** Seemingly unrelated estimations of linear regressions predicting differences between the coefficients of fathers (n = 895) and mothers (n = 1181) for six parenting behaviors

	Affection						Responsivity					
	Fathers^a^		Mothers^a^		Difference tests^b^		Fathers^a^		Mothers^a^		Difference tests^b^	
Predictors	*B*	SE	*B*	SE	*F*	*p*	*B*	SE	*B*	SE	*F*	*p*
*Parental factors*												
Level of education (years)	−.01	.01	.01	.01	2.87	0.09	−.00	.01	.01	.01	0.35	0.55
Age (years)	−.01	.01	−.00	.00	0.45	0.50	−.01	.01	−.01*	.00	0.00	1.00
Working hours	−.00	.00	.00	.00	1.60	0.21	−.00	.00	−.00	.00	1.25	0.26
Working hours partner	.00*	.00	.00	.00	2.09	0.15	.00	.00	−.00	.00	0.30	0.59
*Child factors*												
Gender												
Male (reference category)												
Female	.08	.04	.05	.04	0.16	0.69	.05	.04	.01	.03	0.46	0.50
Age (years)	−.06***	.01	−.03***	.01	6.57	<0.05	−.02**	.01	−.01	.01	1.66	0.20
*Social support, from:*												
Parents/parents-in-law	−.00	.02	.02	.02	0.77	0.38	−.01	.02	.02	.02	1.44	0.23
Other family members	−.06*	.03	−.01	.02	1.81	0.18	−.05	.03	−.00	.02	1.72	0.19
Friends	.06*	.03	−.00	.02	3.84	0.05	.03	.03	−.01	.02	1.36	0.24
Neighbors	−.01	.02	−.03	.02	0.52	0.47	−.02	.03	−.03	.02	0.11	0.74
Child care/playgroup/school	−.02	.02	.02	.02	1.49	0.22	−.01	.02	−.01	.02	0.05	0.82
Constant	6.33***	.20	5.66***	.23			5.96***	.24	5.81***	.23		
*R²*	.22		.11				.06		.04			

	Explaining						Autonomy Support^c^					
	Fathers^a^		Mothers^a^		Difference tests^b^		Fathers^a^		Mothers^a^		Difference tests^b^	
Predictors	*B*	SE	*B*	SE	*F*	*p*	*B*	SE	*B*	SE	*F*	*p*
*Parental factors*												
Level of education (years)	.01	.01	.01	.01	0.01	0.94	.04***	.01	.05***	.01	0.16	0.68
Age (years)	−.00	.01	.00	.01	0.06	0.81	−.01	.01	.00	.01	0.82	0.37
Working hours	−.00	.00	−.00	.00	0.18	0.67	−.00	.00	.01**	.00	3.97	<0.05
Working hours partner	.00	.00	−.00	.00	1.64	0.20	.01***	.00	−.00*	.00	23.08	<0.001
*Child factors*												
Gender												
Male (reference category)												
Female	.02	.04	−.02	.04	0.35	0.56	.06	.05	.02	.05	1.32	0.25
Age (years)	−.02***	.01	−.01	.01	2.33	0.13	.05***	.01	.04***	.01	0.54	0.46
*Social support, from:*												
Parents/parents-in-law	−.04	.03	.00	.02	1.51	0.22	−.03	.03	−.00	.02	0.45	0.50
Other family members	−.01	.03	.01	.02	0.35	0.56	.03	.03	−.02	.03	0.96	0.33
Friends	.01	.03	.01	.02	0.00	0.99	.03	.03	.02	.03	0.04	0.84
Neighbors	.01	.02	−.03	.02	2.20	0.14	.02	.03	−.02	.03	0.89	0.35
Child care/playgroup/school	−.00	.02	.01	.02	0.17	0.68	.03	.03	.04	.03	0.03	0.85
Constant	5.41***	.28	5.31***	.25			3.19***	.33	3.21***	.28		
*R²*	.05		.01				.13		.10			

	Punishment						Rewarding^c^					
	Fathers^a^		Mothers^a^		Difference tests^b^		Fathers^a^		Mothers^a^		Difference tests^b^	
Predictors	*B*	SE	*B*	SE	*F*	*p*	*B*	SE	*B*	SE	*F*	*p*
*Parental factors*												
Level of education (years)	−.03*	.01	−.05**	.02	0.83	0.36	−.04**	.02	−.01	.02	2.02	0.16
Age (years)	−.01	.01	−.02***	.01	1.76	0.18	.01	.01	−.03***	.01	14.53	<0.001
Working hours	−.00	.00	−.00	.00	0.69	0.41	.00	.00	−.01	.00	3.73	0.05
Working hours partner	−.01*	.00	−.00	.00	0.17	0.68	−.00	.00	−.01*	.00	0.85	0.36
*Child factors*												
Gender												
Male (reference category)												
Female	−.08	.07	−.18**	.06	1.23	0.27	−.08	.08	.07	.07	1.94	0.16
Age (years)	−.03***	.01	−.01	.01	2.41	0.12	−.04***	.01	.02	.01	11.05	<0.001
*Social support, from:*												
Parents/parents-in-law	.05	.04	.02	.03	0.37	0.54	.01	.04	.01	.04	0.00	0.97
Other family members	.07	.05	−.03	.03	2.98	0.08	.08	.05	.00	.04	1.11	0.29
Friends	−.06	.04	.06	.03	4.55	<0.05	.01	.05	.03	.04	0.17	0.68
Neighbors	.02	.04	−.02	.03	0.53	0.47	.05	.04	−.01	.04	0.87	0.35
Child care/playgroup/school	−.01	.04	.10**	.03	4.92	<0.05	−.00	.04	.13**	.05	4.02	<0.05
Constant	4.66***	.36	5.18***	.30			3.61***	.44	4.64***	.40		
*R²*	.10		.13				.05		.08			

All values are weighed. All coefficients are unstandardized

^a^Regression results on the two subsamples (fathers and mothers)

^b^Results of the Seemingly Unrelated ESTimation (SUEST) postestimation analyses in which the regression results on the two subsamples were combined in order test for differences in the coefficients between fathers and mothers (Wald chi-squared tests)

^c^Only children aged 2 or older are included fathers (*n* = 853) and mothers (*n* = 1113)

*p < .05. **p < .01. ***p < .001

### Correlates of parenting behaviors: differences between fathers and mothers

In the third step, SUEST analyses were performed in order to test for differences in the coefficients between fathers and mothers. First, overall tests of difference examined whether each model differed across fathers and mothers. The results for the model tests for responsivity (*F* [11, 2,0651] = 0.97, *p* = 0.47), explaining (*F* [11, 2065] = 1.07, *p* = 0.39), and punishment (*F* [11, 2065] = 1.55, *p* = 0.11) were not statistically significant, showing that fathers and mothers did not differ in the way parental factors, child factors, and social support were related to their parenting behavior. For affection (*F* [11, 2065] = 2.82, *p* < 0.01), autonomy support (*F* [11, 1954] = 2.70, *p* < 0.01), and rewarding (*F* [11, 1955] = 2.51, *p* < 0.01), significant differences in the overall test were found, indicating that there were some differences between fathers and mothers.

Subsequently, several pairwise tests were conducted, for each coefficient separately. The results of these analyses are presented in Table 3: columns 4 and 7. In line with our theorizing, some factors were stronger predictors of fathers’ parenting behavior than of mothers’. Both for affection and rewarding, we found age of the child (H2b) to be a stronger predictor for fathers than for mothers. For affection, the older the children, the less affection fathers showed towards their children; this was also true for mothers, but the effect was smaller. For rewarding, the results indicate that fathers made less use of this strategy as the child was older; no such relationship was found for mothers.

We also found that for autonomy support, the number of working hours of the partner (H1d) was a stronger predictor for fathers than for mothers. Namely, the higher the number of working hours of their partners, the more autonomy fathers provided to their children. This was not found for mothers. We also found that the higher the number of working hours of their partners, the more affection fathers showed to their children; no such relation was found for mothers. This difference between fathers and mothers was not statistically significant though.

For other factors, such as age of the parent (H1b), we found differences between fathers and mothers but in the opposite direction of our hypotheses. The older the mothers were, the less they rewarded their children; no such relationship was found for the fathers. We also found that the higher the number of mothers’ working hours (H1c), the more autonomy they provided to their children; no such relationship was found for fathers. And finally, the more support mothers received from the childcare facility, playground, or school (H3), the more they rewarded their children; no such relationship was found for fathers. Two factors were found not to be predictors for differences between fathers’ and mothers’ parenting behavior: the educational level of the parent (H1a) and the gender of the child (H2a).

In conclusion, our results signified that only our hypotheses H1d and H2b—stating that the partner’s number of working hours and the child’s age are stronger predictors of the parenting behavior of fathers than that of mothers—were confirmed, but only for autonomy support (working hours of the partner) and for rewarding and affection (age of the child). All other hypotheses had to be rejected, either for lack of significant differences between fathers and mothers (educational level of the parent (H1a) and child’s gender (H2a)), or because the factors were, contrary to our expectations, stronger predictors for mothers than for fathers (age of the parent (H1b), number of working hours of the parent (H1c), and social support (H3)).

## Discussion

We investigated the contribution of several parent and child characteristics as well as social support to the parenting behavior of fathers and mothers of children aged 2–17. Fathers as well as mothers provided information about their use of a broad range of parenting behaviors, including affection, responsivity, explaining, autonomy, support, rewarding, and punishing. Fathers’ and mothers’ reported use of rewarding, punishment and autonomy support were similar, but mothers rated themselves higher on affection, responsivity, and explaining than fathers did, which is generally in line with previous research (Gryczkowski et al. 2010; Lickenbrock and Braungart-Rieker 2015; Verhoeven et al. 2007).

The main aim of the current study was to investigate whether there were differences between fathers and mothers in the factors associated with their parenting behavior. Based on ample evidence showing that fathers and mothers are socialized to parent differently (Bianchi et al. 2012; Doucet 2009), it was theorized that the social expectations for fathers’ behaviors might be less fixed than for mothers and that fathers are less invested in the parental role than mothers, therefore sensitizing fathers’ parenting behaviors to specific influences and circumstances (Elam et al. 2017).

Our expectation that the parenting behavior of fathers is more dependent on parental factors, child factors, and social support than that of mothers is only partially confirmed by the results of our analysis. In general, many similarities were found. For example, our study suggests that the contribution of education to parenting tends to be the same for fathers and mothers. Both higher-educated fathers and mothers provided their child with more autonomy and punished their child less often than lower-educated parents. These results are consistent with previous studies showing that lower-educated parents use authoritarian parenting strategies more often (Jansen 2009; Trifan et al. 2014). Furthermore, as previous research (Roche et al. 2014; Verhoeven et al. 2012) showed that mothers tend to grant higher levels of autonomy as children grow older, the current study suggests that this holds for fathers too.

Only a few differences were found. For example, the (negative) relationship of children’s age with parental affection and rewarding was stronger for fathers than for mothers, which was in line with our hypothesis. However, these results contradict earlier research, which only found a relationship between the age of children and the parenting of mothers, yet for other parenting strategies (autonomy granting, overcontrol and sensitivity; Laursen et al. 2010; Verhoeven et al. 2012). We also found that the more support mothers receive from formal institutions like childcare, the playgroup, and school, the more they rewarded their child; the same was not found for fathers. This suggests that, given mothers’ greater daily involvement in childcare, formal support is more associated with the parenting of mothers than with the parenting of fathers and that, accordingly, the parenting of mothers is more affected by it (Grolnick et al. 1996; McConnell et al. 2011).

Whereas partners’ number of working hours was found not to be related to mothers’ parenting behavior, we observed a relationship with fathers’ parenting. Controlled for their own number of working hours, fathers granted more autonomy to their children as their partner worked more hours. This is an interesting finding, which probably can be explained by changing value patterns resulting from increased women’s labor market participation. Just as working mothers positively affected the labor market participation of their daughters (Van Putten et al. 2008), the manner in which working mothers reconcile work and care, and parent their children may also set an example for their partners. By demonstrating to their partners that their children can be safely granted more autonomy, working mothers may stimulate their partners to do the same.

Despite these differences, the general picture that emerges from our study is that there were more similarities than differences in fathers’ and mothers’ correlates of parenting, which is in line with previous research (Castillo et al. 2011; Lickenbrock and Braungart-Rieker 2015; Verhoeven et al. 2007). Fathers’ parenting behaviors seem to be related to parent, child and contextual factors in ways that are similar to how these factors are related to mothers’ parenting behaviors, although this might not extend to *all* aspects of parenting and *all* parent, child and contextual factors.

Our expectation that parental characteristics, child characteristics, and social support would exert more influence on the parenting practices of fathers than of mothers was based on our assumption that social expectations for fathers’ behaviors might be less fixed than for mothers. That we found more similarities than differences might be an indication that social expectations for fathers’ behaviors have changed and that, as a result, fathers no longer have more discretion in defining their caring role than mothers. Alternatively, it might be an indication that social expectations for mothers’ behaviors have changed and that, consequently, mothers in time have acquired the same discretion as fathers in defining their caring role. Such a conversion of parenting behaviors of fathers and mothers may be due to changing values concerning the roles of men and women resulting from women’s increasing labor market participation. In the Netherlands, where the current study was conducted and where in the past 30 years women’s labor market participation has increased substantially, support for “traditional” values concerning the roles of women and men in parenting has changed substantially; the vast majority of Dutch men and women now feel that both partners should be enabled to combine care with paid labor (Portegijs and Cloïn 2012).

Our findings are in line with the general evidence on parenting, which suggests that, in many Western countries, differences in fathers’ and mothers’ roles and behaviors are becoming smaller (Cabrera et al. 2014; Fagan et al. 2014). Such an interpretation of our results is consistent with recent literature in which “process” or “ecological” models toward parenting, like those of Belsky (1984) and Bronfenbrenner and Luscher (1995), are revaluated (see for example: Cabrera et al. 2014; Elam et al. 2017). In such an approach the roles of both fathers and mothers seem to be less fixed but, as Cabrera et al. (2014, p. 339) argue, “sometimes fathers will enact roles played by mothers, and vice versa, in response to environmental conditions that require adaptation”.

### Limitations and Future Directions

The findings of our study must be viewed in the context of its limitations. A first limitation is that the results are based on cross-sectional data, hence no causal relationships can be drawn. For example, it is assumed that work demands may influence parenting behaviors, but the relationship may also be the other way round. Childrearing challenges may prevent parents (probably women in particular) from holding jobs with high demands (Roeters et al. 2009). Future research might focus on longitudinal studies to interpret these relationships more fully.

Second, it should be noted that to measure parental behavior only parental self-reports were used. Parents may be giving socially desirable answers and their reports of parental behavior may differ from their actual behavior. However, some studies have addressed this issue and found that what parents reported gave a good indication of their actual behavior (Johnston et al. 2004; Vereijken et al. 1997).

In addition, the effect sizes in our models were relatively small, our data were not nationally representative, and our findings may be less applicable to parents with lower incomes and lower education. Furthermore, due to secondary analyses on an existing data set different measures of social support were only assessed by one item each and a number of relevant factors were not included in the study, like parental and child personality characteristics (Braungart-Rieker et al. 2014; Laxman et al. 2013) and quality of marital relationships (Schoppe-Sullivan and Mangelsdorf 2013; Simons et al. 1990). More research is needed on these and other relevant factors to better understand the differences and similarities between the parenting behaviors of fathers and mothers. Because, as our study showed, mothers’ work demands are connected with paternal parenting behaviors, issues related to reconciliation between work and family are of special interest, like satisfaction with the division of labor (Phares et al. 2009), marital satisfaction and support from the partner (Elam et al. 2017; Planalp et al. 2013).

Finally, our research was carried out in the Netherlands. In the past decades, in the Netherlands, as in most other countries in the Western world, men and women’s roles have become less traditional, with fathers taking a more active role in caring for their children (Merens and Van den Brakel 2014). Future research could investigate to what extent our results can be generalized to countries in which roles for men and women are more traditional.

## Electronic supplementary material

supplementary Materials(DOCX 24 kb)
