# Supplementary material for: Socio-demographic Correlates of Fathers' and Mothers’ Parenting Behaviors
Source: J Child Fam Stud. 2018 Mar 22;27(7):2315–27. doi: 10.1007/s10826-018-1059-7 (PMC5993847; doi:10.1007/s10826-018-1059-7)
Supplement: Supplementary file 1 — supplementary Materials(DOCX 24 kb) [file 10826_2018_1059_MOESM1_ESM.docx]

**Supplementary Materials**

**Affection scale (five items)**

ALIGNMENT OUTPUT

INVARIANCE ANALYSIS

Intercepts/Thresholds

Intercept for AFFECT1 (=item 1 affection)

Group Group Value Value Difference SE P value

1 0 5.450 5.462 -0.012 0.024 0.629

Approximate Measurement Invariance Holds For Groups:

0 1

Weighted Average Value Across Invariant Groups: 5.455

R-square/Explained variance/Invariance index: 0.991

Invariant Group Values, Difference to Average and Significance

Group Value Difference SE P-value

0 5.462 0.007 0.014 0.629

1 5.450 -0.005 0.010 0.629

Intercept for AFFECT2 (=item 2 affection)

Group Group Value Value Difference SE P value

1 0 5.129 5.100 0.029 0.035 0.408

Approximate Measurement Invariance Holds For Groups:

0 1

Weighted Average Value Across Invariant Groups: 5.116

R-square/Explained variance/Invariance index: 1.000

Invariant Group Values, Difference to Average and Significance

Group Value Difference SE P-value

0 5.100 -0.017 0.020 0.408

1 5.129 0.012 0.015 0.408

Intercept for AFFECT3 (=item 3 affection)

Group Group Value Value Difference SE P value

1 0 5.238 5.240 -0.002 0.022 0.923

Approximate Measurement Invariance Holds For Groups:

0 1

Weighted Average Value Across Invariant Groups: 5.239

R-square/Explained variance/Invariance index: 1.000

Invariant Group Values, Difference to Average and Significance

Group Value Difference SE P-value

0 5.240 0.001 0.013 0.923

1 5.238 -0.001 0.010 0.923

Intercept for AFFECT4 (=item 4 affection)

Group Group Value Value Difference SE P value

1 0 3.557 3.522 0.034 0.071 0.631

Approximate Measurement Invariance Holds For Groups:

0 1

Weighted Average Value Across Invariant Groups: 3.542

R-square/Explained variance/Invariance index: 0.979

Invariant Group Values, Difference to Average and Significance

Group Value Difference SE P-value

0 3.522 -0.020 0.041 0.631

1 3.557 0.015 0.031 0.631

Intercept for AFFECT5 (=item 5 affection)

Group Group Value Value Difference SE P value

1 0 5.213 5.343 -0.131 0.035 0.000

Approximate Invariance Was Not Found For This Parameter.

Loadings

Loadings for AFFECT1 (=item 1 affection)

Group Group Value Value Difference SE P value

1 0 0.574 0.629 -0.055 0.039 0.160

Approximate Measurement Invariance Holds For Groups:

0 1

Weighted Average Value Across Invariant Groups: 0.597

R-square/Explained variance/Invariance index: 0.924

Invariant Group Values, Difference to Average and Significance

Group Value Difference SE P-value

0 0.629 0.032 0.022 0.160

1 0.574 -0.024 0.017 0.160

Loadings for AFFECT2 (=item 2 affection)

Group Group Value Value Difference SE P value

1 0 0.833 0.980 -0.148 0.060 0.013

Approximate Measurement Invariance Holds For Groups:

0 1

Weighted Average Value Across Invariant Groups: 0.896

R-square/Explained variance/Invariance index: 0.839

Invariant Group Values, Difference to Average and Significance

Group Value Difference SE P-value

0 0.980 0.084 0.034 0.013

1 0.833 -0.063 0.026 0.013

Loadings for AFFECT3 (=item 3 affection)

Group Group Value Value Difference SE P value

1 0 0.557 0.556 0.000 0.020 0.989

Approximate Measurement Invariance Holds For Groups:

0 1

Weighted Average Value Across Invariant Groups: 0.556

R-square/Explained variance/Invariance index: 1.000

Invariant Group Values, Difference to Average and Significance

Group Value Difference SE P-value

0 0.556 0.000 0.011 0.989

1 0.557 0.000 0.009 0.989

Loadings for AFFECT4 (=item 4 affection)

Group Group Value Value Difference SE P value

1 0 0.599 0.551 0.048 0.081 0.555

Approximate Measurement Invariance Holds For Groups:

0 1

Weighted Average Value Across Invariant Groups: 0.579

R-square/Explained variance/Invariance index: 0.728

Invariant Group Values, Difference to Average and Significance

Group Value Difference SE P-value

0 0.551 -0.027 0.046 0.555

1 0.599 0.021 0.035 0.555

Loadings for AFFECT5 (=item 5 affection)

Group Group Value Value Difference SE P value

1 0 0.573 0.508 0.064 0.042 0.128

Approximate Measurement Invariance Holds For Groups:

0 1

Weighted Average Value Across Invariant Groups: 0.545

R-square/Explained variance/Invariance index: 0.068

Invariant Group Values, Difference to Average and Significance

Group Value Difference SE P-value

0 0.508 -0.037 0.024 0.128

1 0.573 0.028 0.018 0.128

Average Invariance index: 0.836

**Explaining scale (six items)**

ALIGNMENT OUTPUT

INVARIANCE ANALYSIS

Intercepts/Thresholds

Intercept for RESPONS1 (=item 1 responsivity)

Group Group Value Value Difference SE P value

1 0 5.381 5.370 0.011 0.027 0.672

Approximate Measurement Invariance Holds For Groups:

0 1

Weighted Average Value Across Invariant Groups: 5.376

R-square/Explained variance/Invariance index: 0.998

Invariant Group Values, Difference to Average and Significance

Group Value Difference SE P-value

0 5.370 -0.006 0.015 0.672

1 5.381 0.005 0.012 0.672

Intercept for RESPONS2 (=item 2 responsivity)

Group Group Value Value Difference SE P value

1 0 5.387 5.389 -0.001 0.020 0.942

Approximate Measurement Invariance Holds For Groups:

0 1

Weighted Average Value Across Invariant Groups: 5.388

R-square/Explained variance/Invariance index: 1.000

Invariant Group Values, Difference to Average and Significance

Group Value Difference SE P-value

0 5.389 0.001 0.011 0.942

1 5.387 -0.001 0.009 0.942

Intercept for RESPONS3 (=item 3 responsivity)

Group Group Value Value Difference SE P value

1 0 4.983 4.939 0.044 0.027 0.103

Approximate Measurement Invariance Holds For Groups:

0 1

Weighted Average Value Across Invariant Groups: 4.964

R-square/Explained variance/Invariance index: 0.983

Invariant Group Values, Difference to Average and Significance

Group Value Difference SE P-value

0 4.939 -0.025 0.015 0.103

1 4.983 0.019 0.012 0.103

Intercept for RESPONS4 (=item 4 responsivity)

Group Group Value Value Difference SE P value

1 0 4.872 4.882 -0.010 0.024 0.673

Approximate Measurement Invariance Holds For Groups:

0 1

Weighted Average Value Across Invariant Groups: 4.876

R-square/Explained variance/Invariance index: 1.000

Invariant Group Values, Difference to Average and Significance

Group Value Difference SE P-value

0 4.882 0.006 0.013 0.673

1 4.872 -0.004 0.010 0.673

Intercept for RESPONS5 (=item 5 responsivity)

Group Group Value Value Difference SE P value

1 0 4.909 4.938 -0.029 0.024 0.230

Approximate Measurement Invariance Holds For Groups:

0 1

Weighted Average Value Across Invariant Groups: 4.922

R-square/Explained variance/Invariance index: 0.992

Invariant Group Values, Difference to Average and Significance

Group Value Difference SE P-value

0 4.938 0.016 0.014 0.230

1 4.909 -0.012 0.010 0.230

Intercept for RESPONS6 (=item 6 responsivity)

Group Group Value Value Difference SE P value

1 0 5.340 5.347 -0.007 0.021 0.737

Approximate Measurement Invariance Holds For Groups:

0 1

Weighted Average Value Across Invariant Groups: 5.343

R-square/Explained variance/Invariance index: 0.996

Invariant Group Values, Difference to Average and Significance

Group Value Difference SE P-value

0 5.347 0.004 0.012 0.737

1 5.340 -0.003 0.009 0.737

Loadings

Loadings for RESPONS1 (=item 1 responsivity)

Group Group Value Value Difference SE P value

1 0 0.411 0.504 -0.093 0.035 0.008

Approximate Measurement Invariance Holds For Groups:

0 1

Weighted Average Value Across Invariant Groups: 0.451

R-square/Explained variance/Invariance index: 0.735

Invariant Group Values, Difference to Average and Significance

Group Value Difference SE P-value

0 0.504 0.053 0.020 0.008

1 0.411 -0.040 0.015 0.008

Loadings for RESPONS2 (=item 2 responsivity)

Group Group Value Value Difference SE P value

1 0 0.530 0.515 0.015 0.022 0.508

Approximate Measurement Invariance Holds For Groups:

0 1

Weighted Average Value Across Invariant Groups: 0.524

R-square/Explained variance/Invariance index: 0.971

Invariant Group Values, Difference to Average and Significance

Group Value Difference SE P-value

0 0.515 -0.008 0.013 0.508

1 0.530 0.006 0.009 0.508

Loadings for RESPONS3 (=item 3 responsivity)

Group Group Value Value Difference SE P value

1 0 0.651 0.655 -0.005 0.024 0.849

Approximate Measurement Invariance Holds For Groups:

0 1

Weighted Average Value Across Invariant Groups: 0.653

R-square/Explained variance/Invariance index: 0.999

Invariant Group Values, Difference to Average and Significance

Group Value Difference SE P-value

0 0.655 0.003 0.014 0.849

1 0.651 -0.002 0.010 0.849

Loadings for RESPONS4 (=item 4 responsivity)

Group Group Value Value Difference SE P value

1 0 0.742 0.691 0.051 0.030 0.095

Approximate Measurement Invariance Holds For Groups:

0 1

Weighted Average Value Across Invariant Groups: 0.720

R-square/Explained variance/Invariance index: 0.659

Invariant Group Values, Difference to Average and Significance

Group Value Difference SE P-value

0 0.691 -0.029 0.017 0.095

1 0.742 0.022 0.013 0.095

Loadings for RESPONS5 (=item 5 responsivity)

Group Group Value Value Difference SE P value

1 0 0.676 0.656 0.020 0.025 0.412

Approximate Measurement Invariance Holds For Groups:

0 1

Weighted Average Value Across Invariant Groups: 0.668

R-square/Explained variance/Invariance index: 0.964

Invariant Group Values, Difference to Average and Significance

Group Value Difference SE P-value

0 0.656 -0.012 0.014 0.412

1 0.676 0.009 0.011 0.412

Loadings for RESPONS6 (=item 6 responsivity)

Group Group Value Value Difference SE P value

1 0 0.457 0.480 -0.023 0.024 0.357

Approximate Measurement Invariance Holds For Groups:

0 1

Weighted Average Value Across Invariant Groups: 0.467

R-square/Explained variance/Invariance index: 0.960

Invariant Group Values, Difference to Average and Significance

Group Value Difference SE P-value

0 0.480 0.013 0.014 0.357

1 0.457 -0.010 0.011 0.357

Average Invariance index: 0.938

**Explaining scale (five items)**

ALIGNMENT OUTPUT

INVARIANCE ANALYSIS

Intercepts/Thresholds

Intercept for EXPLAI1 (=item 1 explaining)

Group Group Value Value Difference SE P value

1 0 4.890 4.870 0.020 0.034 0.563

Approximate Measurement Invariance Holds For Groups:

0 1

Weighted Average Value Across Invariant Groups: 4.881

R-square/Explained variance/Invariance index: 0.994

Invariant Group Values, Difference to Average and Significance

Group Value Difference SE P-value

0 4.870 -0.011 0.020 0.563

1 4.890 0.009 0.015 0.563

Intercept for EXPLAI2 (=item 2 explaining)

Group Group Value Value Difference SE P value

1 0 4.899 4.783 0.116 0.053 0.028

Approximate Measurement Invariance Holds For Groups:

0 1

Weighted Average Value Across Invariant Groups: 4.849

R-square/Explained variance/Invariance index: 0.905

Invariant Group Values, Difference to Average and Significance

Group Value Difference SE P-value

0 4.783 -0.066 0.030 0.028

1 4.899 0.050 0.023 0.028

Intercept for EXPLAI3 (=item 3 explaining)

Group Group Value Value Difference SE P value

1 0 5.201 5.328 -0.127 0.040 0.002

Approximate Measurement Invariance Holds For Groups:

0 1

Weighted Average Value Across Invariant Groups: 5.255

R-square/Explained variance/Invariance index: 0.229

Invariant Group Values, Difference to Average and Significance

Group Value Difference SE P-value

0 5.328 0.072 0.023 0.002

1 5.201 -0.055 0.017 0.002

Intercept for EXPLAI4 (=item 4 explaining)

Group Group Value Value Difference SE P value

1 0 5.252 5.272 -0.020 0.030 0.504

Approximate Measurement Invariance Holds For Groups:

0 1

Weighted Average Value Across Invariant Groups: 5.260

R-square/Explained variance/Invariance index: 0.989

Invariant Group Values, Difference to Average and Significance

Group Value Difference SE P-value

0 5.272 0.011 0.017 0.504

1 5.252 -0.009 0.013 0.504

Intercept for EXPLAI5 (=item 5 explaining)

Group Group Value Value Difference SE P value

1 0 5.391 5.391 -0.001 0.023 0.977

Approximate Measurement Invariance Holds For Groups:

0 1

Weighted Average Value Across Invariant Groups: 5.391

R-square/Explained variance/Invariance index: 0.996

Invariant Group Values, Difference to Average and Significance

Group Value Difference SE P-value

0 5.391 0.000 0.013 0.977

1 5.391 0.000 0.010 0.977

Loadings

Loadings for EXPLAI1 (=item 1 explaining)

Group Group Value Value Difference SE P value

1 0 0.410 0.425 -0.015 0.040 0.713

Approximate Measurement Invariance Holds For Groups:

0 1

Weighted Average Value Across Invariant Groups: 0.416

R-square/Explained variance/Invariance index: 0.951

Invariant Group Values, Difference to Average and Significance

Group Value Difference SE P-value

0 0.425 0.008 0.023 0.713

1 0.410 -0.006 0.017 0.713

Loadings for EXPLAI2 (=item 2 explaining)

Group Group Value Value Difference SE P value

1 0 0.580 0.558 0.022 0.053 0.678

Approximate Measurement Invariance Holds For Groups:

0 1

Weighted Average Value Across Invariant Groups: 0.570

R-square/Explained variance/Invariance index: 0.798

Invariant Group Values, Difference to Average and Significance

Group Value Difference SE P-value

0 0.558 -0.013 0.030 0.678

1 0.580 0.009 0.023 0.678

Loadings for EXPLAI3 (=item 3 explaining)

Group Group Value Value Difference SE P value

1 0 0.520 0.439 0.082 0.053 0.120

Approximate Measurement Invariance Holds For Groups:

0 1

Weighted Average Value Across Invariant Groups: 0.485

R-square/Explained variance/Invariance index: 0.000

Invariant Group Values, Difference to Average and Significance

Group Value Difference SE P-value

0 0.439 -0.047 0.030 0.120

1 0.520 0.035 0.023 0.120

Loadings for EXPLAI4 (=item 4 explaining)

Group Group Value Value Difference SE P value

1 0 0.446 0.447 -0.001 0.034 0.967

Approximate Measurement Invariance Holds For Groups:

0 1

Weighted Average Value Across Invariant Groups: 0.447

R-square/Explained variance/Invariance index: 0.999

Invariant Group Values, Difference to Average and Significance

Group Value Difference SE P-value

0 0.447 0.001 0.019 0.967

1 0.446 -0.001 0.015 0.967

Loadings for EXPLAI5 (=item 5 explaining)

Group Group Value Value Difference SE P value

1 0 0.421 0.475 -0.053 0.046 0.248

Approximate Measurement Invariance Holds For Groups:

0 1

Weighted Average Value Across Invariant Groups: 0.444

R-square/Explained variance/Invariance index: 0.761

Invariant Group Values, Difference to Average and Significance

Group Value Difference SE P-value

0 0.475 0.030 0.026 0.248

1 0.421 -0.023 0.020 0.248

Average Invariance index: 0.762

**Autonomy Support scale (six items)**

ALIGNMENT OUTPUT

INVARIANCE ANALYSIS

Intercepts/Thresholds

Intercept for AUTO1 (=item 1 autonomy support)

Group Group Value Value Difference SE P value

1 0 4.758 4.880 -0.122 0.060 0.044

Approximate Measurement Invariance Holds For Groups:

0 1

Weighted Average Value Across Invariant Groups: 4.811

R-square/Explained variance/Invariance index: 0.000

Invariant Group Values, Difference to Average and Significance

Group Value Difference SE P-value

0 4.880 0.069 0.034 0.044

1 4.758 -0.053 0.026 0.044

Intercept for AUTO2 (=item 2 autonomy support)

Group Group Value Value Difference SE P value

1 0 4.977 4.891 0.086 0.048 0.076

Approximate Measurement Invariance Holds For Groups:

0 1

Weighted Average Value Across Invariant Groups: 4.939

R-square/Explained variance/Invariance index: 0.129

Invariant Group Values, Difference to Average and Significance

Group Value Difference SE P-value

0 4.891 -0.049 0.027 0.076

1 4.977 0.037 0.021 0.076

Intercept for AUTO3 (=item 3 autonomy support)

Group Group Value Value Difference SE P value

1 0 3.877 3.724 0.153 0.071 0.030

Approximate Measurement Invariance Holds For Groups:

0 1

Weighted Average Value Across Invariant Groups: 3.811

R-square/Explained variance/Invariance index: 0.119

Invariant Group Values, Difference to Average and Significance

Group Value Difference SE P-value

0 3.724 -0.087 0.040 0.030

1 3.877 0.066 0.031 0.030

Intercept for AUTO4 (=item 4 autonomy support)

Group Group Value Value Difference SE P value

1 0 4.093 4.117 -0.024 0.018 0.174

Approximate Measurement Invariance Holds For Groups:

0 1

Weighted Average Value Across Invariant Groups: 4.103

R-square/Explained variance/Invariance index: 0.000

Invariant Group Values, Difference to Average and Significance

Group Value Difference SE P-value

0 4.117 0.014 0.010 0.174

1 4.093 -0.010 0.008 0.174

Intercept for AUTO5 (=item 5 autonomy support)

Group Group Value Value Difference SE P value

1 0 4.691 4.385 0.306 0.058 0.000

Approximate Invariance Was Not Found For This Parameter.

Intercept for AUTO6 (=item 6 autonomy support)

Group Group Value Value Difference SE P value

1 0 3.587 3.663 -0.076 0.063 0.231

Approximate Measurement Invariance Holds For Groups:

0 1

Weighted Average Value Across Invariant Groups: 3.620

R-square/Explained variance/Invariance index: 0.000

Invariant Group Values, Difference to Average and Significance

Group Value Difference SE P-value

0 3.663 0.043 0.036 0.231

1 3.587 -0.033 0.027 0.231

Loadings

Loadings for AUTO1 (=item 1 autonomy support)

Group Group Value Value Difference SE P value

1 0 0.650 0.597 0.053 0.054 0.329

Approximate Measurement Invariance Holds For Groups:

0 1

Weighted Average Value Across Invariant Groups: 0.627

R-square/Explained variance/Invariance index: 0.689

Invariant Group Values, Difference to Average and Significance

Group Value Difference SE P-value

0 0.597 -0.030 0.031 0.329

1 0.650 0.023 0.023 0.329

Loadings for AUTO2 (=item 2 autonomy support)

Group Group Value Value Difference SE P value

1 0 0.342 0.398 -0.056 0.049 0.249

Approximate Measurement Invariance Holds For Groups:

0 1

Weighted Average Value Across Invariant Groups: 0.366

R-square/Explained variance/Invariance index: 0.000

Invariant Group Values, Difference to Average and Significance

Group Value Difference SE P-value

0 0.398 0.032 0.028 0.249

1 0.342 -0.024 0.021 0.249

Loadings for AUTO3 (=item 3 autonomy support)

Group Group Value Value Difference SE P value

1 0 0.631 0.587 0.045 0.059 0.449

Approximate Measurement Invariance Holds For Groups:

0 1

Weighted Average Value Across Invariant Groups: 0.612

R-square/Explained variance/Invariance index: 0.727

Invariant Group Values, Difference to Average and Significance

Group Value Difference SE P-value

0 0.587 -0.025 0.033 0.449

1 0.631 0.019 0.026 0.449

Loadings for AUTO4 (=item 4 autonomy support)

Group Group Value Value Difference SE P value

1 0 0.751 0.755 -0.004 0.043 0.925

Approximate Measurement Invariance Holds For Groups:

0 1

Weighted Average Value Across Invariant Groups: 0.753

R-square/Explained variance/Invariance index: 0.993

Invariant Group Values, Difference to Average and Significance

Group Value Difference SE P-value

0 0.755 0.002 0.024 0.925

1 0.751 -0.002 0.018 0.925

Loadings for AUTO5 (=item 5 autonomy support)

Group Group Value Value Difference SE P value

1 0 0.608 0.830 -0.223 0.060 0.000

Approximate Invariance Was Not Found For This Parameter.

Loadings for AUTO6 (=item 6 autonomy support)

Group Group Value Value Difference SE P value

1 0 0.649 0.642 0.007 0.044 0.869

Approximate Measurement Invariance Holds For Groups:

0 1

Weighted Average Value Across Invariant Groups: 0.646

R-square/Explained variance/Invariance index: 0.979

Invariant Group Values, Difference to Average and Significance

Group Value Difference SE P-value

0 0.642 -0.004 0.025 0.869

1 0.649 0.003 0.019 0.869

Average Invariance index: 0.364

**Punishment scale (six items)**

ALIGNMENT OUTPUT

INVARIANCE ANALYSIS

Intercepts/Thresholds

Intercept for PUNIS1 (=item 1 punishment)

Group Group Value Value Difference SE P value

1 0 2.945 3.073 -0.128 0.078 0.098

Approximate Measurement Invariance Holds For Groups:

0 1

Weighted Average Value Across Invariant Groups: 3.000

R-square/Explained variance/Invariance index: 0.000

Invariant Group Values, Difference to Average and Significance

Group Value Difference SE P-value

0 3.073 0.073 0.044 0.098

1 2.945 -0.055 0.033 0.098

Intercept for PUNIS2 (=item 2 punishment)

Group Group Value Value Difference SE P value

1 0 3.922 3.796 0.127 0.082 0.122

Approximate Measurement Invariance Holds For Groups:

0 1

Weighted Average Value Across Invariant Groups: 3.868

R-square/Explained variance/Invariance index: 0.326

Invariant Group Values, Difference to Average and Significance

Group Value Difference SE P-value

0 3.796 -0.072 0.047 0.122

1 3.922 0.054 0.035 0.122

Intercept for PUNIS3 (=item 3 punishment)

Group Group Value Value Difference SE P value

1 0 4.805 4.805 0.000 0.030 1.000

Approximate Measurement Invariance Holds For Groups:

0 1

Weighted Average Value Across Invariant Groups: 4.805

R-square/Explained variance/Invariance index: 1.000

Invariant Group Values, Difference to Average and Significance

Group Value Difference SE P-value

0 4.805 0.000 0.017 1.000

1 4.805 0.000 0.013 1.000

Intercept for PUNIS4 (=item 4 punishment)

Group Group Value Value Difference SE P value

1 0 3.356 3.357 -0.002 0.047 0.973

Approximate Measurement Invariance Holds For Groups:

0 1

Weighted Average Value Across Invariant Groups: 3.356

R-square/Explained variance/Invariance index: 1.000

Invariant Group Values, Difference to Average and Significance

Group Value Difference SE P-value

0 3.357 0.001 0.027 0.973

1 3.356 -0.001 0.020 0.973

Intercept for PUNIS5 (=item 5 punishment)

Group Group Value Value Difference SE P value

1 0 1.691 1.842 -0.151 0.054 0.005

Approximate Measurement Invariance Holds For Groups:

0 1

Weighted Average Value Across Invariant Groups: 1.756

R-square/Explained variance/Invariance index: 0.000

Invariant Group Values, Difference to Average and Significance

Group Value Difference SE P-value

0 1.842 0.086 0.031 0.005

1 1.691 -0.065 0.023 0.005

Intercept for PUNIS6 (=item 6 punishment)

Group Group Value Value Difference SE P value

1 0 3.744 3.671 0.073 0.065 0.259

Approximate Measurement Invariance Holds For Groups:

0 1

Weighted Average Value Across Invariant Groups: 3.713

R-square/Explained variance/Invariance index: 0.456

Invariant Group Values, Difference to Average and Significance

Group Value Difference SE P-value

0 3.671 -0.042 0.037 0.259

1 3.744 0.031 0.028 0.259

Loadings

Loadings for PUNIS1 (=item 1 punishment)

Group Group Value Value Difference SE P value

1 0 1.032 0.994 0.037 0.069 0.590

Approximate Measurement Invariance Holds For Groups:

0 1

Weighted Average Value Across Invariant Groups: 1.016

R-square/Explained variance/Invariance index: 0.000

Invariant Group Values, Difference to Average and Significance

Group Value Difference SE P-value

0 0.994 -0.021 0.039 0.590

1 1.032 0.016 0.030 0.590

Loadings for PUNIS2 (=item 2 punishment)

Group Group Value Value Difference SE P value

1 0 0.874 0.985 -0.111 0.094 0.239

Approximate Measurement Invariance Holds For Groups:

0 1

Weighted Average Value Across Invariant Groups: 0.922

R-square/Explained variance/Invariance index: 0.338

Invariant Group Values, Difference to Average and Significance

Group Value Difference SE P-value

0 0.985 0.063 0.054 0.239

1 0.874 -0.048 0.041 0.239

Loadings for PUNIS3 (=item 3 punishment)

Group Group Value Value Difference SE P value

1 0 0.425 0.440 -0.015 0.035 0.669

Approximate Measurement Invariance Holds For Groups:

0 1

Weighted Average Value Across Invariant Groups: 0.431

R-square/Explained variance/Invariance index: 0.690

Invariant Group Values, Difference to Average and Significance

Group Value Difference SE P-value

0 0.440 0.008 0.020 0.669

1 0.425 -0.006 0.015 0.669

Loadings for PUNIS4 (=item 4 punishment)

Group Group Value Value Difference SE P value

1 0 0.827 0.694 0.132 0.092 0.152

Approximate Measurement Invariance Holds For Groups:

0 1

Weighted Average Value Across Invariant Groups: 0.770

R-square/Explained variance/Invariance index: 0.000

Invariant Group Values, Difference to Average and Significance

Group Value Difference SE P-value

0 0.694 -0.075 0.053 0.152

1 0.827 0.057 0.040 0.152

Loadings for PUNIS5 (=item 5 punishment)

Group Group Value Value Difference SE P value

1 0 0.501 0.551 -0.051 0.058 0.382

Approximate Measurement Invariance Holds For Groups:

0 1

Weighted Average Value Across Invariant Groups: 0.523

R-square/Explained variance/Invariance index: 0.395

Invariant Group Values, Difference to Average and Significance

Group Value Difference SE P-value

0 0.551 0.029 0.033 0.382

1 0.501 -0.022 0.025 0.382

Loadings for PUNIS6 (=item 6 punishment)

Group Group Value Value Difference SE P value

1 0 0.940 0.862 0.078 0.077 0.308

Approximate Measurement Invariance Holds For Groups:

0 1

Weighted Average Value Across Invariant Groups: 0.907

R-square/Explained variance/Invariance index: 0.000

Invariant Group Values, Difference to Average and Significance

Group Value Difference SE P-value

0 0.862 -0.044 0.044 0.308

1 0.940 0.034 0.033 0.308

Average Invariance index: 0.350

**Rewarding scale (six items)**

ALIGNMENT OUTPUT

INVARIANCE ANALYSIS

Intercepts/Thresholds

Intercept for REWAR1 (=item 1 rewarding)

Group Group Value Value Difference SE P value

1 0 2.856 3.033 -0.177 0.061 0.004

Approximate Measurement Invariance Holds For Groups:

0 1

Weighted Average Value Across Invariant Groups: 2.932

R-square/Explained variance/Invariance index: 0.465

Invariant Group Values, Difference to Average and Significance

Group Value Difference SE P-value

0 3.033 0.100 0.035 0.004

1 2.856 -0.077 0.026 0.004

Intercept for REWAR2 (=item 2 rewarding)

Group Group Value Value Difference SE P value

1 0 3.218 3.238 -0.021 0.028 0.466

Approximate Measurement Invariance Holds For Groups:

0 1

Weighted Average Value Across Invariant Groups: 3.226

R-square/Explained variance/Invariance index: 0.936

Invariant Group Values, Difference to Average and Significance

Group Value Difference SE P-value

0 3.238 0.012 0.016 0.466

1 3.218 -0.009 0.012 0.466

Intercept for REWAR3 (=item 3 rewarding)

Group Group Value Value Difference SE P value

1 0 2.617 2.542 0.075 0.049 0.130

Approximate Measurement Invariance Holds For Groups:

0 1

Weighted Average Value Across Invariant Groups: 2.584

R-square/Explained variance/Invariance index: 0.000

Invariant Group Values, Difference to Average and Significance

Group Value Difference SE P-value

0 2.542 -0.042 0.028 0.130

1 2.617 0.032 0.021 0.130

Intercept for REWAR4 (=item 4 rewarding)

Group Group Value Value Difference SE P value

1 0 3.689 3.733 -0.044 0.038 0.250

Approximate Measurement Invariance Holds For Groups:

0 1

Weighted Average Value Across Invariant Groups: 3.708

R-square/Explained variance/Invariance index: 0.843

Invariant Group Values, Difference to Average and Significance

Group Value Difference SE P-value

0 3.733 0.025 0.022 0.250

1 3.689 -0.019 0.017 0.250

Intercept for REWAR5 (=item 5 rewarding)

Group Group Value Value Difference SE P value

1 0 5.025 4.813 0.212 0.054 0.000

Approximate Invariance Was Not Found For This Parameter.

Intercept for REWAR6 (=item 6 rewarding)

Group Group Value Value Difference SE P value

1 0 5.642 5.407 0.235 0.031 0.000

Approximate Invariance Was Not Found For This Parameter.

Loadings

Loadings for REWAR1 (=item 1 rewarding)

Group Group Value Value Difference SE P value

1 0 1.006 0.943 0.064 0.080 0.425

Approximate Measurement Invariance Holds For Groups:

0 1

Weighted Average Value Across Invariant Groups: 0.979

R-square/Explained variance/Invariance index: 0.482

Invariant Group Values, Difference to Average and Significance

Group Value Difference SE P-value

0 0.943 -0.036 0.045 0.425

1 1.006 0.028 0.035 0.425

Loadings for REWAR2 (=item 2 rewarding)

Group Group Value Value Difference SE P value

1 0 0.946 0.926 0.020 0.067 0.766

Approximate Measurement Invariance Holds For Groups:

0 1

Weighted Average Value Across Invariant Groups: 0.937

R-square/Explained variance/Invariance index: 0.791

Invariant Group Values, Difference to Average and Significance

Group Value Difference SE P-value

0 0.926 -0.011 0.038 0.766

1 0.946 0.009 0.029 0.766

Loadings for REWAR3 (=item 3 rewarding)

Group Group Value Value Difference SE P value

1 0 0.866 0.798 0.068 0.073 0.354

Approximate Measurement Invariance Holds For Groups:

0 1

Weighted Average Value Across Invariant Groups: 0.837

R-square/Explained variance/Invariance index: 0.419

Invariant Group Values, Difference to Average and Significance

Group Value Difference SE P-value

0 0.798 -0.038 0.041 0.354

1 0.866 0.029 0.032 0.354

Loadings for REWAR4 (=item 4 rewarding)

Group Group Value Value Difference SE P value

1 0 0.983 0.998 -0.015 0.066 0.823

Approximate Measurement Invariance Holds For Groups:

0 1

Weighted Average Value Across Invariant Groups: 0.989

R-square/Explained variance/Invariance index: 0.000

Invariant Group Values, Difference to Average and Significance

Group Value Difference SE P-value

0 0.998 0.008 0.037 0.823

1 0.983 -0.006 0.029 0.823

Loadings for REWAR5 (=item 5 rewarding)

Group Group Value Value Difference SE P value

1 0 0.464 0.554 -0.090 0.062 0.147

Approximate Measurement Invariance Holds For Groups:

0 1

Weighted Average Value Across Invariant Groups: 0.503

R-square/Explained variance/Invariance index: 0.000

Invariant Group Values, Difference to Average and Significance

Group Value Difference SE P-value

0 0.554 0.051 0.035 0.147

1 0.464 -0.039 0.027 0.147

Loadings for REWAR6 (=item 6 rewarding)

Group Group Value Value Difference SE P value

1 0 0.094 0.100 -0.006 0.030 0.831

Approximate Measurement Invariance Holds For Groups:

0 1

Weighted Average Value Across Invariant Groups: 0.097

R-square/Explained variance/Invariance index: 0.000

Invariant Group Values, Difference to Average and Significance

Group Value Difference SE P-value

0 0.100 0.004 0.017 0.831

1 0.094 -0.003 0.013 0.831

Average Invariance index: 0.394
